# Acute Cerebellitis and Obstructive Hydrocephalus: An Unseen Neurological Complication After Surgical Repair for Tetralogy of Fallot

**DOI:** 10.7759/cureus.62355

**Published:** 2024-06-14

**Authors:** Anupam Das

**Affiliations:** 1 Cardiothoracic Vascular Surgery, All India Institute of Medical Sciences, Jodhpur, Jodhpur, IND

**Keywords:** management of obstructive hydrocephalus, neurological infections, cerebellar-ataxia, tetralogy of fallot (tof), cerebellitis

## Abstract

Acute cerebellitis with obstructive hydrocephalus post-Tetralogy of Fallot surgery is extremely rare but can present aggressively in pediatric cases. Early diagnosis is critical for prompt medical and surgical intervention. We report a fatal case in a 7-year-old boy post-surgery, where neurological symptoms rapidly progressed, leading to drowsiness and intermittent response to commands. Despite initial computed tomography scans showing no abnormality, subsequent scans revealed cerebellitis and hydrocephalus. Treatment with steroids, antibiotics, and cerebrospinal fluid drainage was unsuccessful, and the condition’s etiology remained unclear despite negative serological tests and cultures. This highlights the challenge of diagnosing and treating acute cerebellitis, especially when no specific cause is found and when deterioration is swift. The role of opioids in pediatric patients and their potential association with neurosurgical complications is also discussed, prompting further inquiry into postoperative symptoms and opioid-related risks in susceptible individuals.

## Introduction

Acute cerebellitis is a rare inflammatory entity but is a serious cause of cerebellar ataxia in the pediatric population [1,2] and its presentation varies from benign symptoms to fulminant fatal presentation with posterior fossa compression, hydrocephalus, or intracranial hypertension [2]. Contrast-enhanced CT (CECT) and magnetic resonance imaging (MRI) of the brain are both good modalities for establishing the diagnosis; however, MRI is better when establishing the etiology of the condition, which is often a medical and surgical emergency. Its presentation in a patient of Tetralogy of Fallot (TOP) in the postoperative period is very unusual and hence is being reported for future reference.

## Case presentation

A 7-year-old boy was admitted for the surgical repair of TOF. History was remarkable for cyanosis for 1 year of age and easy fatigability for 2 years. There was no specific history of seizures/cyanotic spells/loss of consciousness/prolonged noncardiac illness. Neurological examination was unremarkable. Computed tomography (CT) angiography disclosed TOF with severe infundibular and valvular stenosis with good-sized and confluent branch pulmonary arteries. Laboratory investigations revealed a hematocrit of 63.6 with normal total (TLC) and differential leucocyte count (DLC).

The repair performed by aorto-bicaval cannulation under moderate hypothermia (28°C) included autologous pericardial patch closure of the ventricular septal defect with infundibular resection with a transannular patch with autologous pericardium. He was weaned off cardiopulmonary bypass (CPB) with inotropic support of adrenaline 0.05 µg/kg/minute and milrinone 0.5 µg/kg/minute. The CPB duration was 155 minutes and the ischemic arrest time was 120 minutes. The child had type II heart block with an intrinsic ventricular rate of 30-40 beats/minute. The pRV/LV (ratio of peak right ventricular to peak left ventricular pressure) was 0.6 and the postoperative transoesophageal echocardiography showed no residual shunt, no tricuspid regurgitation, mild pulmonary regurgitation with normal biventricular function. After extubation on the same day (day 0), his postoperative course in the pediatric intensive care unit was uneventful and he was kept on epicardial atrioventricular sequential pacing with temporary pacemaker on oral enalapril 0.2 mg/kg/day in two divided doses, combination of oral furosemide and spironolactone each 1 mg/kg/day once daily, oral orciprenaline 10 mg twice daily and intravenous dexamethasone 0.15 mg/kg/day 8 hourly. As per the institutional protocol he was given injections of cefoperazone-sulbactam and amikacin for the first 48 hours after the surgery. Orciprenaline and dexamethasone were started for type II heart block. For the first 2 days, the child also received fentanyl infusion for analgesia at a dose of 1 µg/kg/hour.

**Figure 1:**
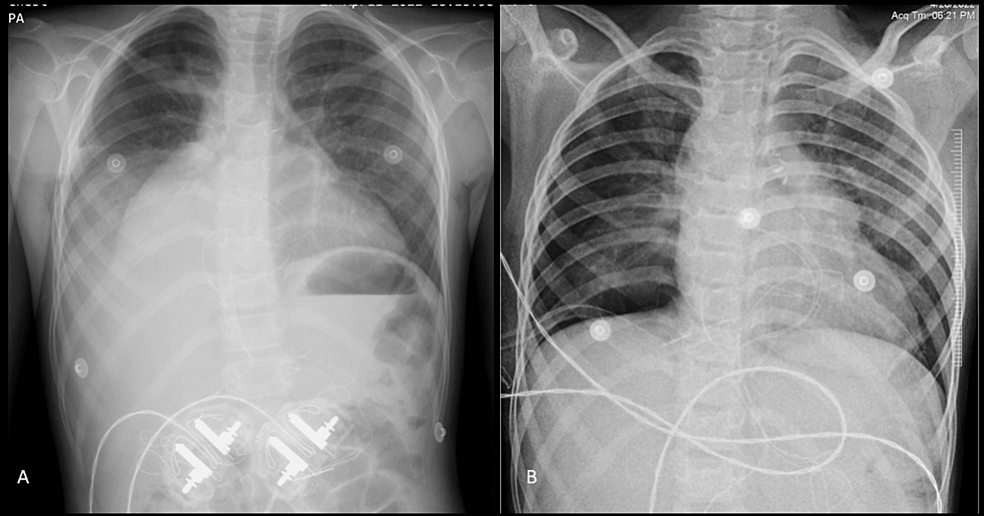
Day 4 chest X-ray showing significant right-sided pleural effusion (A); chest X-ray after right ICD insertion on day 5 (B) ICD: intercostal drainage

The chest radiograph on day 4 revealed a right-sided pleural effusion (Figure 1A).

**Figure 2:**
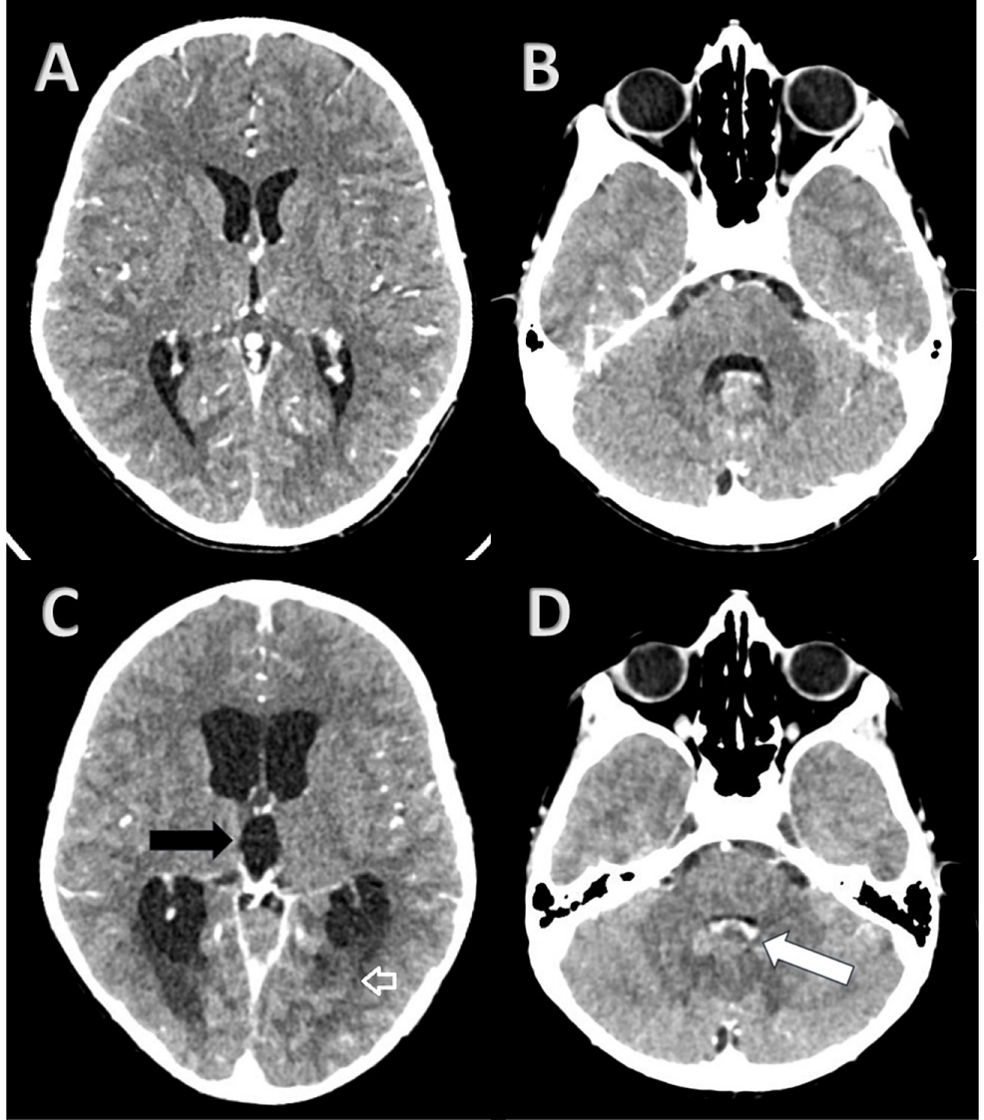
Sequential axial CECT brain scans—day 5 Normal size of ventricles and cerebellum in A and B; day 6: decompensated hydrocephalus (black arrow in figure C) with periventricular ooze (white arrow in figure C) secondary to bulky and edematous cerebellum causing effacement of fourth ventricle (white arrow in figure D) suggestive of cerebellitis.

He continued to have TLC in the range of 12,500-13,000/µL with predominantly neutrophilia (85-92%) with no clinical signs of infection (day 3). On day 5, he developed irritability, headache and ataxia. Dexamethasone was increased to 6 hourly doses and he underwent a CECT scanning of the brain which was normal (Figures 2A-2B). 

The child was also started on an injection of levetiracetam. A right intercostal drainage (ICD) tube was inserted (Figure 1B) with drainage of 400 ml serous fluid which was sent for cytology and biochemistry which were unremarkable. Spironolactone was withdrawn in view of suspicion of spironolactone-induced ataxia. On day 6, he became drowsy with intermittent response to verbal commands, there was no neck rigidity, fever, hyper/hyporeflexia, or hyper/hypotonia. Hemodynamics remained stable. MRI brain could not be planned in view of the need for continuous pacing, as an alternative CECT brain was repeated with CT angiography of brain and neck vessels which revealed an edematous bulky cerebellum with the inferior part of vermis partially effacing fourth ventricle with upstream ventricular dilatation with periventricular ooze suggestive of decompensated hydrocephalus (Figures 2C-2D). Mannitol was added to the treatment in the dose of 1 g/kg as an intravenous infusion over 30 minutes 6 hourly. In view of deteriorating neurological status, he was intubated immediately and external ventricular drain (EVD) was inserted bedside. Cerebrospinal fluid was found to be clear in appearance, cytology was paucicellular, with few erythrocytes and occasional lymphomononuclear cells, protein levels were 12 mg/dL (normal range 15-45 mg/dL) and glucose levels were 121 mg/dL (normal 40-70 mg/dL). Injection meropenem and vancomycin were added along with methylprednisolone pulse therapy. Gram staining and fungal smear of CSF didn’t yield any pus cells or organisms. He developed 103 °F fever and his TLC levels rose to 18,900/µL. CRP rose to 56.7 mg/L, serum ferritin increased to 611 ng/mL, and IL6 rose to 21.5 pg/mL with slightly raised procalcitonin levels (0.59, normal ≤0.1 ng/mL). On day 7, the child further deteriorated with decerebrate posturing despite adequate drainage of CSF via the EVD with continuous fever and finally succumbed to progressively deteriorating neurological condition. Blood and urine aerobic and fungal cultures were later followed up and were found to be negative. CSF viral PCR were negative for CMV (cytomegalovirus), Japanese encephalitis, adenovirus, Ebstein Barr virus, human herpes virus 7, human herpes virus 6, human parechovirus, parvovirus B19, human simplex virus 1 & 2 and coronavirus. Pre-operative evaluation of COVID-19 (Coronavirus disease-19) was found to be negative; however, a striking finding in the serum reports was raised levels of COVID-19 IgG antibodies (Table 1).

**Table 1:** Blood and CSF investigations CSF: cerebrospinal fluid; TLC: total leucocyte count; CRP: C-reactive protein; CMV: cytomegalovirus; HHV: human herpesvirus; HSV: herpes simplex virus

Investigations	Measured Value	Reference Value
CSF cytology	Paucicellular, few erythrocytes, occasional lymphomononuclear cells	
CSF protein	12 mg%	15-45 mg%
CSF glucose	121 mg%	40-70 mg%
TLC	18,900/µL	4000-11,000/µL
CRP	56.7 mg/L	≤ 5 mg/L
Serum ferritin	611 ng/mL	30-400 ng/mL
Serum procalcitonin	0.59 ng/mL	≤0.01 mg/mL
Serum IL 6	21.5 pg/mL	<5 pg/mL
CSF PCR		
CMV	Negative	
HHV 7	Negative	
HHV 6	Negative	
Parvovirus B19	Negative	
Varicella zoster	Negative	
Ebstein Barr	Negative	
Parechovirus	Negative	
Adenovirus	Negative	
Enteroviruses	Negative	
HSV 1	Negative	
HSV 2	Negative	
Serum SARS-COV-2 IgG antibodies	83.21 U/mL	<0.80 U/mL

Cerebellitis due to COVID-19 has not been reported in the literature but it must be emphasized that it could not be excluded with certainty in the absence of a pathological autopsy. The etiology of acute cerebellitis, hence, remained obscured in our case and further investigations could not be attempted due to the rapid downhill course of the child eventually leading to his death.

## Discussion

To the best of the literature search, acute cerebellitis with obstructive hydrocephalus has not been reported after surgery for TOF. Many cases of acute cerebellitis have been encountered but only a few cases with fulminant course, as in our case have been reported [3].

Etiology includes postinfectious (rotavirus, Epstein-Barr virus, varicella zoster, herpes simplex virus, mumps, coxsackie virus, influenza, human herpes virus, West Nile virus, streptococcal infections, salmonella, mycoplasma, dengue fever, malaria, toxoplasmosis), post-immunization (diphtheria, pertussis, tetanus, varicella, influenza), autoimmune disorders like lupus [1,4]. There is no consensus regarding the optimal medical treatment yet, but systemic corticosteroids, intravenous immunoglobulins, antimicrobials [4], antiepileptic medications and osmotic diuretics (mannitol, furosemide) form important constituents of multispectral treatment of this entity [1].

As with our case, most of the cases reported in the literature could not yield any specific etiology and the rapid progression of this disease didn’t allow us to investigate the other pathogens as mentioned earlier. The plan for a ventriculoperitoneal shunt in our case could not be contemplated due to the rapid deterioration. Due to the decompensated nature of the presentation and rapid downhill course, along with medical measures including anti-edema treatment, the pediatric neurology and neurosurgery teams decided on the surgical drainage at the earliest. Intravenous immunoglobulins were not immediately available; however, escalation of antibiotics was done immediately. This experience has added to the spectrum of neurological complications post-cardiac surgery, the earliest diagnosis of which is very much emphasized because of the fulminant course leading to death. The use of opioids in the pediatric population has also been linked to neurosurgical complications [5]. The presence of raised levels of COVID-19 antibodies raised the suspicion that the child may have been previously exposed to the virus.

## Conclusions

This undiagnosed case of cerebellitis wherein the etiology could not be elicited, raised a few questions regarding the postoperative period: (1) Did the neutrophilia and higher TLC counts (usually expected post-cardiac surgery) with no clinical evidence of infection on day 3 should have been investigated in more detail? (2) Can the use of opioids in postoperative pain relief and sedation be attributed to such a condition in susceptible patients? Furthermore, the association of COVID-19 to neurological symptoms could not be established but having mentioned so, it could not have been ruled out without a pathological autopsy, which the parents didn’t consent to. However, this study emphasized the fact that any neurological event after any cardiac surgery must be evaluated at the earliest and an index of suspicion for cerebellitis (even though very rare post-cardiac surgery) should be high based on clinical symptoms.
